# Efficacy of the complementary therapies in the management of cancer pain in palliative care: A systematic review*

**DOI:** 10.1590/1518-8345.4213.3377

**Published:** 2020-09-30

**Authors:** Luís Carlos Lopes-Júnior, Gabriela Sylvestre Rosa, Raphael Manhães Pessanha, Sara Isabel Pimentel de Carvalho Schuab, Karolini Zuqui Nunes, Maria Helena Costa Amorim

**Affiliations:** 1 Universidade Federal do Espírito Santo, Departamento de Enfermagem, Vitória, ES, Brazil.; 2 Universidade Federal de São Paulo, Escola Paulista de Enfermagem, São Paulo, SP, Brazil.

**Keywords:** Complementary Therapies, Adult, Cancer Pain, Palliative Care, Oncology Nursing, Evidence-Based Nursing, Terapias Complementares, Adulto, Dor do Câncer, Cuidados Paliativos, Enfermagem Oncológica, Enfermagem Baseada em Evidências, Terapias Complementarias, Adulto, Dolor en Cáncer, Cuidados Paliativos, Enfermería Oncológica, Enfermería Basada en la Evidencia

## Abstract

**Objective:**

to synthesize the knowledge and to critically evaluate the evidences arising from randomized controlled trials on the efficacy of the complementary therapies in the management of cancer pain in adult patients with cancer in palliative care.

**Method:**

a systematic review guided by the Preferred Reporting Items for Systematic Reviews and Meta-Analyses. The search for articles in the MEDLINE, ISI *Web of Knowledge*, CENTRAL Cochrane, and PsycINFO databases, as well as the manual search, selection of studies, data extraction, and methodological assessment using the Cochrane Bias Risk tool were performed independently by two reviewers.

**Results:**

eight hundred and fifteen (815) studies were identified, six of them being selected and analyzed, of which three used massage therapy, one study used a combination of progressive muscle relaxation and guided imaging, and another two studies used acupuncture. Most of the studies had an uncertain risk of bias (n=4; 67%).

**Conclusion:**

while the evidence from the studies evaluating the use of massage therapy or the use of progressive muscle relaxation and guided imaging for the management of cancer pain in these patients demonstrated significant benefits, the other two studies that evaluated the use of acupuncture as a complementary therapy showed contradictory results, therefore, needing more research studies to elucidate such findings.

## Abstract (pt)

**Objetivo:**

sintetizar o conhecimento e avaliar criticamente as evidências provenientes de ensaios clínicos controlados randomizados sobre a eficácia das terapias complementares no manejo da dor oncológica em pacientes adultos com câncer em cuidados paliativos.

**Método:**

revisão sistemática guiada pelo *Preferred Reporting Items for Systematic Reviews and Meta-Analyses.* A busca dos artigos nas bases de dados MEDLINE, ISI *Web of Knowledge*, CENTRAL Cochrane e PsycINFO, bem como a busca manual, seleção dos estudos, extração dos dados e avaliação metodológica pela ferramenta do Risco de Viés da Cochrane foram realizadas por dois revisores de forma independente.

**Resultados:**

foram identificados 815 estudos, sendo seis selecionados e analisados, dos quais três utilizaram a massagem terapêutica, um estudo usou uma combinação de relaxamento muscular progressivo e imagem guiada, e outros dois estudos a acupuntura. A maioria dos estudos apresentou risco de viés incerto (n=4; 67%).

**Conclusão:**

enquanto as evidências dos estudos que avaliaram o uso da massagem terapêutica ou o uso de relaxamento muscular progressivo e imagem guiada para manejo da dor oncológica nesses pacientes mostraram benefícios significativos, os outros dois estudos que avaliaram o uso da acupuntura como terapia complementar exibiram resultados divergentes, necessitando, portanto, de mais pesquisas para elucidar tais achados.

## Abstract (es)

**Objetivo:**

sintetizar conocimiento y realizar el análisis crítico de las evidencias procedentes de ensayos clínicos controlados aleatorios sobre la eficacia de las terapias complementarias en el manejo del dolor oncológico en pacientes adultos con cáncer en cuidados paliativos.

**Método:**

revisión sistemática guiada por el *Preferred Reporting Items for Systematic Reviews and Meta-Analyses.* La búsqueda de artículos en las bases de datos MEDLINE, ISI *Web of Knowledge*, CENTRAL Cochrane y PsycINFO, así como la búsqueda manual, selección de estudios, extracción de datos y evaluación metodológica con la utilización de la herramienta Riesgo de Sesgo de Cochrane se realizaron por dos revisores de forma independiente.

**Resultados:**

se identificaron 815 estudios, de los cuales, seis fueron seleccionados y analizados, siendo que tres utilizaron masaje terapéutico, un estudio utilizó una combinación de relajación muscular progresiva e imagen guiada, y otros dos estudios la acupuntura. La mayor parte de los estudios presentaron riesgo de sesgo incierto (n=4; 67%)

**Conclusión:**

mientras las evidencias de los estudios que evalúan el uso de la del masaje terapéutico o el uso de la relajación muscular progresiva y las imágenes guiadas para el manejo del dolor oncológico en estos pacientes demostraron beneficios significativos, los otros dos estudios que evaluaron el uso de la acupuntura como terapia complementaria mostraron resultados divergentes, razón por la cual se requiere de más investigación para dilucidar tales hallazgos.

## Introduction

The latest report on the global cancer burden in the world, according to the GLOBOCAN 2018 estimates, has estimated about 18.1 million new cases of cancer and 9.6 million deaths due to malignant neoplasms in 2018^(^ 1 ^)^. Reaching alarming levels, cancer is a contemporary global public health problem, being the second leading cause of mortality in several countries^(^ 2 ^)^. Estimates from the World Health Organization (WHO) indicate that, in 2030, cancer will reach approximately 27 million incident cases worldwide, 17 million deaths, and 75 million people with annual diagnosis^(^ 3 ^)^. The greatest effect will be noticeable in low- and middle-income countries. For each year of the 2020-2022 triennium, in Brazil the occurrence of 625 thousand new cancer cases was estimated^(^ 4 ^)^.

Cancer pain is a symptom related to multiple factors, defined as “simultaneous sensations of acute and chronic pain, of different levels of intensity, associated with the invasive spread of tumor cells in the body; a consequence of the cancer treatment, including chemotherapy, or cancer-related conditions; being generally described as imprecise, hurting, frightening or as an unbearable sensation, with episodes of intense sensations, accompanied by difficulties to sleep, irritability, depression, suffering, isolation, hopelessness, and helplessness”^(^ 5 ^)^. Although the WHO Analgesic Scale has been widely used^(^ 6 ^-^ 7 ^)^, approximately 40% to 50% of the cancer pain cases have inadequaterelief due to their multi-factorial nature^(^ 8 ^)^. There is still a shortage of effective pain management schemes for many cancer patients, especially those in palliative care^(^ 9 ^-^ 10 ^)^. Thus, a combination of pharmacological and non-pharmacological treatment modalities for cancer pain should be the standard care, due to the complexity of this symptom^(^ 10 ^-^ 11 ^)^.

Palliative care was defined in 1990 and redefined in 2002 by the WHO as an approach that improves the quality of life of patients and their families facing the problem associated with life-threatening illness, through the prevention and relief of suffering by means of early identification and impeccable assessment and treatment of pain and other physical, psychosocial, and spiritual problems^(^ 12 ^)^. Nurses play an important role in palliative care, with responsibility for providing information, counseling, and education to the patients and their families in maintaining the home/hospital dyad^(^ 13 ^)^. Due to the strong bond with patients and for being at the frontline of care, they are in the best position for handling the cancer symptom clusters^(^ 13 ^-^ 15 ^)^. It is highlighted that, for many cancer patients in palliative care, drug therapy is insufficient for pain relief or does not match the patient’s choice^(^ 11 ^)^. Thus, it becomes essential to use complementary therapies (CTs) in addition to the conventional ones for cancer pain management^(^ 11 ^,^ 15 ^-^ 16 ^)^.

The National Center for Complementary and Alternative Medicine (NCCAM) defines Complementary Alternative Medicine as a set of practices, medical and health care systems for individuals who are not considered part of conventional medicine^(^ 17 ^)^. The CTs cover techniques aimed at prevention, promotion, treatment, and recovery, in order to integrate the physical, mental, and spiritual dimensions of the human being. There are several ways to classify these therapies. The NCCAM categorizes them mainly as: use of natural products; body and mind practices; and body-based manipulation practices^(^ 17 ^)^. Over the past three decades, the use of CTs has increased considerably both in pediatric patients^(^ 18 ^-^ 22 ^)^ and in the adult population^(^ 23 ^-^ 26 ^)^. However, the efficacy of the CTs for cancer pain management in adults with cancer in palliative care is still a gap in the scientific literature^(^ 11 ^)^.

In this sense, this study aimed to synthesize the knowledge and to critically evaluate the evidence from randomized controlled trials on the efficacy of the complementary therapies in the management of cancer pain in adult cancer patients in palliative care.

## Method

This study is a systematic review of the literature, which was guided by the Preferred Reporting Items for Systematic Reviews and Meta-Analyses (PRISMA). In order to guarantee the reliability of the data and methodological transparency, we filed the registration in the International Prospective Register of Systematic Reviews (PROSPERO/NHS) – Record Number: CRD42020156074.

To formulate the objective and the review question, the following strategy was used: PICOS (*P – Population or Patients; I – Intervention; C – Comparison; O – Outcomes; S – Study design*), where P = Population (adults with cancer in palliative care), I = Intervention (complementary therapies), C = Comparison (control group not receiving intervention or receiving standard/usual clinical care), O = Outcomes (reduction of cancer pain), and S = Study design (randomized controlled trials)^(^ 27 ^)^. This strategy facilitated the structuring of critical reasoning on the topic and the formulation of the following question: “What is the existing scientific evidence from the randomized controlled trials on the efficacy of complementary therapies in the management of cancer pain in adults with cancer in palliative care?”

Primary studies were included whose design was a randomized controlled trial (RCT) conducted with adult patients (≥ 19 years old), of both genders, diagnosed with any type of malignancy in palliative care; studies covering the efficacy of some complementary therapy classified by The National Center for Complementary and Alternative Medicine (National Institutes of Health, USA), which categorizes them mainly as: use of natural products; body and mind practices; and body-based manipulation practices^(^ 17 ^)^ and whose primary outcome was cancer pain. There was no restriction regarding the languages or publication year. Quasi-experimental studies, literature review studies; theses and dissertations; book chapters, clinical guidelines, technical reports and editorials were excluded. The search for the studies was carried out systematically in four electronic databases: MEDLINE - Medical Literature Analysis and Retrieval System Online (via PubMed), Cochrane Central Register of Controlled Trials (CENTRAL Cochrane), ISI Web of Knowledge via Web of Science, and PsycINFO (Psychology Information).

The search strategy of the studies was composed by a combination of controlled descriptors (indexers in the respective databases) and keywords, according to the indication offered in each electronic database. Thus, to search for articles on MEDLINE, controlled descriptors were used from the Medical Subject Headings (MeSH); and the PsycINFO Thesaurus was consulted for the PsycINFO database. The keywords were established after a thorough reading related to the investigated topic. In order to expand the search strategy, a combination of controlled descriptors and keywords was performed using Boolean operators^(^ 28 ^)^.

The Boolean operators AND and OR were used to obtain restrictive and additive combinations, respectively. In addition, the search was carried out using identified descriptors and with a broader sense, without using the database filters to preserve significant samples and ensure less risk of losses. This strategy justifies the small number of studies selected in view of the sample obtained, added to the fact that we establish the RCT as an inclusion criteria as a design to encompass the strongest evidence for decision-making into clinical practice^(^ 28 ^)^. Figure 1 shows the final search strategy processed in the respective databases.

**Figure 1:** Database search strategy in the MEDLINE/PubMed, CENTRAL Cochrane, ISI Web of Knowledge/Web of Science, and PsycINFO databases, on August 30^th^, 2019. Vitória, ES, Brazil. 2019

Database	Search Strategy
MEDLINE*/PubMed 08-30-2019^¶^	**#1** (("Adult" [MeSH Terms]^†^ AND "Cancer Patients" OR "Advanced Cancer Patients" AND "Neoplasms" [MeSH Terms]^†^OR "Cancer" AND "Palliative Care" [MeSH Terms]^†^ OR "Palliative Medicine" [MeSH Terms]^†^ OR "Hospices" [MeSH]^†^)) **#2** (("Complementary Therapies" [MeSH Terms]^†^ OR "Therapies, Complementary" [All Fields] OR "Complementary Medicine" [All Fields] OR "Alternative Medicine" [All Fields]" OR "Alternative Therapies" [All Fields] OR "Non-pharmacological Interventions" [All Fields])) **#3** (("Cancer Pain" [MeSH Terms]^†^ OR "Cancer-Associated Pain" [All Fields] OR "Cancer-Related Pain" [All Fields] OR "Neoplasm Related Pain" [All Fields] OR "Tumor Associated Pain" [All Fields] OR "Oncological Pain" [All Fields] OR "Oncology Pain" [All Fields])) **#4** (("Randomized Controlled Trial" [MeSH Terms]^†^ OR "Controlled Clinical Trial" [MeSH Terms]^†^ OR "Clinical Trial" [All Fields])) **#5** #1 AND #2 AND #3 AND #4
CENTRAL Cochrane^‡^ 08-30-2019^¶^	**#1** (("Adult" [MeSH Terms]^†^ AND "Cancer Patients" OR "Advanced Cancer Patients" AND "Neoplasms" [MeSH Terms]^†^ OR "Cancer" AND "Palliative Care" [MeSH Terms]^†^ OR "Palliative Medicine" [MeSH Terms]^†^ OR "Hospices" [MeSH]^†^)) **#2** (("Complementary Therapies" [MeSH Terms]^†^ OR "Therapies, Complementary" [All Fields] OR "Complementary Medicine" [All Fields] OR "Alternative Medicine" [All Fields]" OR "Alternative Therapies" [All Fields] OR "Non-pharmacological Interventions" [All Fields])) **#3** (("Cancer Pain" [MeSH Terms]^†^ OR "Cancer-Associated Pain" [All Fields] OR "Cancer-Related Pain" [All Fields] OR "Neoplasm Related Pain" [All Fields] OR "Tumor Associated Pain" [All Fields] OR "Oncological Pain" [All Fields] OR "Oncology Pain" [All Fields])) **#4** (("Randomized Controlled Trial" [MeSH Terms]^†^ OR "Controlled Clinical Trial" [MeSH Terms]^†^ OR "Clinical Trial" [All Fields])) **#5** #1 AND #2 AND #3 AND #4
ISI of Knowledge/Web of Science 08-30-2019^¶^	(TS^||^=("Adult" AND "Cancer Patients" OR "Advanced Cancer Patients" AND "Neoplasms" OR "Cancer" AND "Palliative Care" OR "Palliative Medicine" OR "Hospices") AND TS=("Complementary Therapies" OR "Therapies, Complementary" OR "Complementary Medicine" OR "Alternative Medicine" OR "Alternative Therapies" OR "Non-pharmacological Interventions") AND TS=("Cancer Pain" OR "Cancer-Associated Pain" "OR "Cancer-Related Pain" OR "Neoplasm Related Pain" OR "Tumor Associated Pain" OR "Oncological Pain" OR "Oncology Pain") AND TS=("Randomized Controlled Trial" OR "Controlled Clinical Trial" OR "Clinical Trial" OR "Random Allocation" OR "Double-blind Method" OR "Single-blind Method"))
PsycINFO^§^ 08-30-2019^¶^	(("Neoplasms" [Thesaurus] OR "Oncology" [Thesaurus] OR "Terminal Cancer" [Thesaurus] AND "Palliative Care" [Thesaurus] OR "Terminally Ill Patients" [Thesaurus] OR "Hospice" [Thesaurus] AND "Alternative Medicine" [Thesaurus] OR "Mind Body Therapy" [Thesaurus] OR "Meditation" [Thesaurus] OR "Medicinal Herbs and Plants" [Thesaurus] OR "Massage" [Thesaurus] OR "Hypnotherapy" [Thesaurus] OR "Holistic Health" [Thesaurus] OR "Dietary Supplements" [Thesaurus] OR "Acupuncture" [Thesaurus] OR "Aromatherapy" [Thesaurus] OR "Faith Healing "[Thesaurus] OR "Complementary Therapies" OR "Non-pharmacological interventions" AND "Pain Management" [Thesaurus] OR "Oncological Pain)) AND (("Clinical Trials" [Thesaurus] OR "Randomized Clinical Trials" [Thesaurus] OR "Randomized Controlled Trial" OR "Controlled Clinical Trial"))

* MEDLINE = Medical Literature Analysis and Retrieval System Online;

† MeSH = Medical Subject Headings;

‡ CENTRAL = Cochrane Central Register of Controlled Trials;

§ PsycINFO = Psychology Information

|| TS = Topic;

¶ 08-30-2019= Date the search strategy was carried out

It should be noted that there were no publication date or language restrictions in the search strategy held. In addition to the aforementioned electronic databases, secondary searches were carried out from other sources, such as Clinical Trial Records sites like ClinicalTrials.gov (National Institutes of Health, NIH, USA), and The Brazilian Clinical Trials Registry (via the ReBEC Platform). Moreover, the list of final references contained in the included primary studies was analyzed manually in order to find relevant studies to be added. In this stage of the review, the EndNote™ reference manager (https://www.myendnoteweb.com/) was used to store, organize, and delete duplicates, in order to ensure a systematic, comprehensive, and manageable search.

The sample was independently and blindly selected by two reviewers in August 2019. After this selection, a third reviewer was responsible for analyzing and deciding (together with the previous ones) on the inclusion or exclusion of each article, especially in relation to those containing conflicting decisions. After the selection of the third reviewer, a manual search was conducted based on the references of the selected articles.

Data were extracted based on pre-established tools^(^ 29 ^-^ 31 ^)^ and included four domains: I) Identification of the study, with data such as the title of the article, impact factor of the journal, country of the authors of the study, year of publication, host institution of the study (hospital; University; research facility; multicenter study or study in a single institution); conflicts of Interest; funding; II) Methodological characteristics (study design; objective of the study or research question or hypotheses); sample characteristics, for example, sample size, age, baseline characteristics for the experimental and control groups, recruitment method, drop-outs, follow-up time, statistical analysis; III) Main findings and implications for clinical practice; and IV) Conclusions.

For data extraction, two Microsoft Word^®^ tables were created independently by two researchers to synthesize data from the included studies. After this phase, the tables were compiled into a single one to proceed with the qualitative analyses.

The evaluation of the methodological quality of the studies was defined as an essential process to establish internal validity, checking the possible biases and the reliability of the identified evidence. In this systematic review of RCT, the methodological quality of the included studies was assessed by two independent reviewers, using the Cochrane Bias Risk tool from the Handbook of the Cochrane Collaboration for Systematic Intervention Reviews, version 5.1.0^(^ 32 ^)^, which assesses the following seven domains: I) Allocation of the randomization sequence (selection bias); II) Allocation concealment (selection bias); III) Blinding of the participants and the team involved (performance bias); IV) Blinding of outcome evaluators (detection bias); V) Incomplete outcomes (attrition bias); VI) Report of selective outcome (publication bias); and VII) Other sources of bias. Based on these assessed domains, the studies are classified as low, high, or uncertain bias risk.

The studies were classified according to the risk of bias as follows: “Low” if all the main domains were classified as “low risk”; “Uncertain” if one or two main domains were classified as “uncertain risk”; and “high” if more than two main domains have been classified as “uncertain” or “high risk”. When no information was available, we assign “uncertain risk”^(^ 33 ^)^.

As most of the studies evaluated showed significant methodological differences, it was decided to perform a qualitative synthesis of the data in this systematic review.

## Results

**Figure 2:**
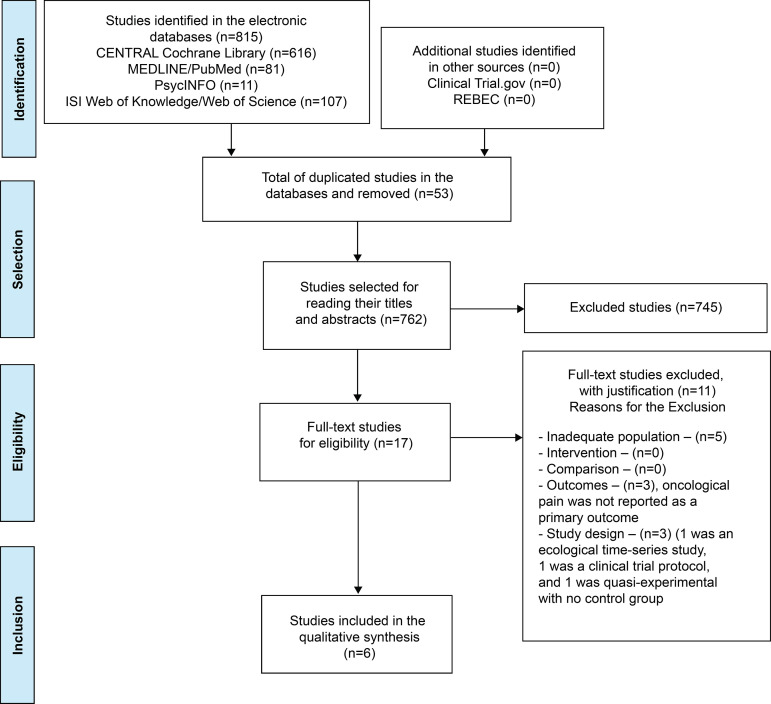
PRISMA flow chart^(^ 34 ^)^ for selecting the studies. Vitória, ES, Brazil. 2019

The searches in the four electronic databases, as well as the manual search in other sources, resulted in 815 studies. We identified 53 studies that were duplicated in the databases. After removing them using the EndNote*™* reference manager*,* 762 studies went on to the selection process by title and abstract. At this stage, 745 studies were excluded for not meeting the pre-established inclusion criteria. The exclusion by title and abstract resulted in the selection of 17 studies that were read in full-text. After this stage for exhaustive reading of the full-text studies, another 11 studies were excluded, resulting, therefore, in six articles that were included for qualitative synthesis and analysis (Figure 2).

Regarding the characteristics of the studies, the publication date of the six articles included varied in the range from 2004 to 2019^(^ 35 ^-^ 40 ^)^, and all were published in the English language with a randomized controlled trial design carried out in different countries.

Figure 3 chronologically synthesizes the main characteristics of the studies included in the qualitative synthesis of this systematic review.

**Figure 3:** Characteristics of the studies included in the systematic review. Vitória, ES, 2019

Reference/Country/	Objective	Randomization & Blinding	Protocol	Intervention		Instruments	Outcomes		Main Results
				Experimental Group (EG)^†^	Control Group (CG)*		Primary	Secondary	
Soden, et al. 2004^(^ 35 ^)^ United Kingdom	To compare the effects of a 4-week aromatherapy massage and massage only on physical and psychological symptoms in patients with advanced cancer	**Randomization:** The treatment allocation was concealed by a numbered opaque envelope and opened after the initial evaluation was completed **Blinding:** The researchers who analyzed the data were blinded to the interventions. The patients who received the massages were not informed about the oils used	The two experimental groups (EGs)^†^(aromatherapy group and massage group) received a 30-minute back massage weekly for four weeks. Lavender essential oil (LEO^§^) was chosen due to its sedative and analgesic effects The LEO^§^ was mixed with sweet almond oil (an inert carrier oil) for a 1% dilution. The patients in the Control Group (CG)* completed the assessment scales weekly during the study period, but did not receive any massage	- EG^†^1) Aromatherapy Group (n=16): massage with LEO and an inert carrier oil - EG^†^2) Massage Group (n=13): massage only with an inert carrier oil	- CG*(n=13): patients who did not receive any massage	- VAS^||^ - Modified Tursky Pain Descriptors Scale - VSH^¶^ Sleep Scale - HADS** - RSCL^††^	Changes in the VAS^||^ scores for pain, from the baseline to the final assessment	Sleep; depression and anxiety	There was a statistically significant reduction in the VAS^||^ pain scores in the aromatherapy (*p* = 0.03) and combined massage (*p* = 0.01) groups after the second treatment. There were no significant changes in the scale of the pain descriptors, and no cumulative analgesic effect over time
Kutner, et al. 2009^(^ 36 ^)^ USA	To assess the efficacy of the massage in reducing pain and suffering symptoms in order to improve the quality of life of patients with advanced cancer	**Randomization:** The individuals were randomized from a central unit by two researchers. The randomization sequence was stratified in blocks and generated by the SAS computer program **Blinding:** The data collectors were blinded to the interventions. The entire study team, except the study coordinators at the study locus and the two researchers at the center, were blinded to the randomization sequence	- EG^†^: The massage intervention included light/smooth *effleurage* (65% of the time), petrissage and release of myofascial trigger point (35% of the time). The most massaged areas of the body were the neck and upper back (nearly 80% of the time) and the arms, hands, legs, and feet (nearly 75% of the time) Inflammation/Infection, hyperesthesia, injury, surgery, catheters, deep vein thrombosis and tumors were avoided. 50% of the sessions were held with the patient in the supine position; 25% in a seated position, and 25% were divided between lying and prone positions. The massage was performed by massage therapists, with at least 6 months of experience in treating patients with advanced cancer - The CG* received "simple touch", which consisted of placing both hands on the participant for 3 minutes: neck base, scapulas, lumbar region, gastrocnemius, heels, clavicles, arms, hands, patellas, and feet. The pressure was light and consistent, with no hand movements from side to side. The control treatments were provided by individuals with no previous experience	- EG^†^: Massage Therapy Group (n=188): Patients receiving massage therapy	- CG*(n=192): Patients receiving simple touch	- MPAC^‡‡^ - BPI^§§^ - MQOL^||||^ - MSAS^¶¶^	Immediate and sustained change in pain levels	Immediate secondary outcomes included mood, heart rate, and respiratory rate. Sustained effects included quality of life, physical and emotional distress, and use of painkillers	Both groups showed an immediate improvement in pain (EG^†^ = -1.87 points (CI: -2.07; -1.67), CG*= -0.97 points (CI: -1,18; -0,76); immediate mood improvement (EG^†^ = 1.58 points (CI: 1.40; 1.76), CG* = 0.97 points (CI: 0.78; 1.16). The EG^†^ obtained a higher score for pain and mood outcomes (p < 0.001). There were no differences between the means of the groups over time in pain (Mean BPI = 0.07 (CI: -0.23; 0.37), quality of life (general QoL = 0.08 (CI: -0,37; 0.53), distress of the symptoms (MSAS Index=-0.002 (CI: -0,12; 0.12) or use of analgesics = -0.10 (CI: -0.25; 0.05)
Lopez-Sendín, et al. 2011^(^ 37 ^)^ Spain	To determine the effects of physical therapy, including massage therapy and exercise, on pain and mood in patients with advanced terminal cancer	**Randomization:** A table of random numbers was generated by computer, created before the study started **Blinding:** A therapist who collected all the study results was blinded to the group interventions	- The physiotherapy intervention in the EG^†^ consisted of several different massage techniques: *Effleurage, petrissage*, and strain/counterstrain techniques over the sensitive points. The patients received passive mobilization, active assistance or resistance exercises, and local and global resistance exercises, in addition to proprioceptive neuromuscular facilitation applied to the tense/painful joints and muscles - The CG* received a "simple touch" (ideal condition for false control), which was applied to areas of pain and maintained for the same period as in the EG^†^. The treated areas included the lower cervical area, shoulder, interscapular area, heels, dorsal region of the foot, and gastrocnemius. In both groups, the risk areas (location of tumors, catheters, surgery) were avoided. All the patients received 6 sessions lasting 30 to 35 minutes over a period of two weeks	EG^†^= Intervention group (n=12): Patients receiving physical therapy, including massage therapy and exercises	CG* (n=12): Patients receiving simple manual touch	- BPI^§§^ - MPAC^‡‡^ - MSAS^¶¶^	Changes in pain levels	Differences in mood levels in the pre-/post-intervention	A significant group x time interaction was found with improvements in the EG^†^ for the BPI index^§§^ (F = 13.2, *p*<0.001), and for the psychological MSAS (F = 8.480, *p* = 0.001). In summary, the present study demonstrated that the combination of massage and exercise can reduce pain and improve mood in patients with terminal cancer
Lam, et al., 2017^(^ 38 ^)^ China and Hong Kong	To test the safety of *si guanxue* acupuncture for cancer pain management	**Randomization:** A computer program was used to randomize the participants. The study coordinator allocated the randomization codes that indicated the study arms in the numbered and sealed envelopes. These envelopes were sealed for the investigators **Blinding:** neither the researchers nor the participants were blinded. Only those who conducted the analyses were blinded	The EG^†^1 only used *si guanxue*, while the EG^†^2 used *si guanxue* in combination with a set of commonly used acupuncture points: Neiguan (PC6), Zusanli (ST36), and Sanyinjiao (SP6) These points also constituted the CG*. Disposable acupuncture needles (0.25 x 25 mm or 0.30 x 40 mm) were inserted under the skin at a vertical depth of 10 to 20 mm. Afterwards, a reinforcement reduction method was used to activate the Qi until the sensation of the arrival of the Qi (numbness, fullness, and weight) was reported by the patients. The patients were kept in dorsal supine with the needles left in place for 30 minutes. The acupuncture treatment consisted of 7 sessions, performed daily or on alternate days	EG^†^_1_) Treatment Arm 1 (n=14): *si guan xue* EG^†^_2_) Treatment Arm 2 (n=14): *si guan xue* plus the commonly used acupuncture points (PC6; ST36; SP6)	CG* (n=14): Patients receiving acupuncture at the commonly used points (PC6; ST36; SP6)	- NRS*** - PGIC^†††^ - EORTC QLQ-C30^‡‡‡^ - KPS^§§§^	Relief of cancer pain and subjective improvement of the patients	Quality of Life	The analysis showed that the reduction in cancer pain in the EG2 was more prominent on day 5 when compared to the control arm (*p*<0.05). There was no difference in the PGIC^†††^, EORTC QLQ-C30^‡‡‡^ or KPS^§§§^ scores among the three groups (*p*>0.05). In addition, no serious adverse events were observed. The use and addition of *si guanxue* in the acupuncture treatment for cancer pain was also considered feasible and manageable
Kim, et al. 2018^(^ 39 ^)^ Republic of Korea	To determine the viability and to evaluate the effects and safety of intradermal acupuncture (IA) in patients who were receiving analgesics for cancer pain	**Randomization:** The block randomization method was used by a statistician to generate random numbers, using the R program. Opaque envelopes numbered and containing the randomization sequences were kept in a safe place **Blinding:** Only the clinician who administered the IA therapy^|||||^ was not blinded to the interventions. The subjects, the outcome evaluators, and the statistician who performed data analysis were blindedto the allocation of the treatment throughout the study	- In the EG^†^, the patients received IA treatment for 3 weeks at the specified acupuncture points (CV12, bilateral ST25, LI4, LR3, PC06, and additionally 0-3 Ashi points). The acupuncture points were selected by consensus of a committee of specialists composed of professors/researchers specialized in traditional Korean Medicine. Disposable, sterile, stainless steel IA needles, measuring 0.18 x 1.3 x 1.5 mm, were fixed with adhesive tape. Each IA needle was kept in the skin for 48 to 72 hours, and all the patients were instructed to press all locations on the needle with their hands 2x/day - In the CG* (Sham IA), all the interventions were the same as for the EG^†^, including issuing the same instructions. However, the tip of the needle has been bent so as to cause a stinging sensation, imitating real acupuncture, without actually piercing the skin. The EG^†^ and CG* interventions were performed by Korean physicians with at least 3 years of clinical experience	EG^†^= IA treatment (n=15): Patients receiving AI	CG*=Sham IA Treatment (n=15): Patients receiving Sham IA	- NRS*** - EORTC QLQ-C30^‡‡‡^	Change in the grade and dosage of analgesics for cancer pain between baseline assessments and after the treatment	Pain intensity and quality of life	Nine patients (64%) in the EG^†^(IA^|||||^) and 5 (38%) in the CG*(Sham IA^|||||^) responded to the three-week intervention. Self-reported pain decreased by -1.54 ± 1.45 and by -1.15 ± 1.57 in the IA^|||||^ and Sham IA^|||||^ groups, respectively, with a reported improvement in quality of life (*p* = 0.017)
De Paolis, et al. 2019^(^ 40 ^)^ Italy	To assess the adjuvant effect of PMR-IGI**** in pain relief in a sample of terminal cancer patients in palliative care	**Randomization:** The patients were allocated using a stratified randomization procedure based on their baseline pain score, which was associated with a randomization list placed in a sealed envelope which was opened by the clinical research nurse (CRN)^¶¶¶^ **Blinding:** There was no blinding	The study had 4 phases: T0, T1, T2 and T3: - T0 (patient registration): Patients admitted to the hospital for at least 48 hours; examined by some CRN^¶¶¶^ - T1 (within 24 hours from T0): Collecting information at the baseline. The EG patients^†^ (group A) were scheduled to an individual PMR-IGI session. The CG* (group B) received the usual care - T2 (within 1h from T1): Each PMR-IGI**** lasted 20 minutes. In the first 4 minutes, a state of psychophysical relaxation was induced by prolonged deep breathing and relaxation of the main muscle groups. The patient was invited by the professional to focus on their voice, tone and volume - T3 (within 2h from the intervention): The patients were reassessed by the ESAS-r^††††^ - the number of acute pain episodes that occurred in the 24-hour period after the intervention and the administration of rescue analgesics were recorded in the CRF^||||||||^	EG^†^=Group A (n=46): Patients receiving intervention (PMR-IGI****)	CG*=Group B (n=45): Patients receiving standard care (without intervention)	- ESAS-r^††††^ - TSDS^‡‡‡‡^	The primary endpoint was a Pain Intensity Difference (PID^§§§§^) score, i.e., a difference in the pain reported before and after the intervention ≥ 1	- Total score for distress, anxiety, and depression; - Number of acute pain episodes reported within 24 hours after the PMR-IGI session****; - Need for rescue painkillers	The Pain Intensity Difference (NRS*** at T3-NRS*** at T1) was 1.83 in the EG (group A) and 0.55 in the CG* (group B), having statistical significance (p<0.0001). The mean total distress score decreased by 8.83 in the EG^†^, and by 1.84 in the CG*. The mean difference in the ESAS-r^††††^ emotional scores (anxiety and depression) was 2.93 in the EG^†^ (p<0.0001) and 0.07 in the CG* (p>0.05)

* CG = Control Group;

† EG = Experimental Group;

§ LEO = Lavender Essential Oil;

|| VAS = Visual Analogue Scale;

¶ VSH - Verran and Snyder-Halpern Sleep Scale;

** HADS = Hospital Anxiety and Depression Scale;

†† RSCL = Rotterdam Symptom Checklist;

‡‡ MPAC = Memorial Pain Assessment Card;

§§ BPI = Brief Pain Inventory;

|||| MQOL = McGill Quality of Life Questionnaire;

¶¶ MSAS = Memorial Symptom Assessment Scale;

*** NRS = Numerical Rating Scale;

††† PGIC = Patient Global Impression of Change;

‡‡‡ EORTC QLQ-C30 = European Organization for Research and Treatment of Cancer Quality of Life Questionnaire;

§§§ KPS = Karnofsky Performance Status Scale;

||||| IA = Intradermal acupuncture;

¶¶¶ CRN = Clinical Research Nurse;

**** PMR-IGI = Progressive Muscle Relaxation (PMR) and Interactive Guided Imagery (IGI);

†††† ESAS-r = Edmonton Symptom Assessment System Revised;

‡‡‡‡ TSDS = Total Symptom Distress Score;

§§§§ PID = Pain Intensity Difference;

|||||||| CRF = Case Report Form

The total number of research participants among the included studies was 609 patients, with samples varying from 24 to 380 patients. Regarding the use of complementary therapies embraced in the included studies, it was verified that three studies used massage therapy^(^ 35 ^-^ 37 ^)^, one study used a combination of progressive muscle relaxation and guided imaging^(^ 40 ^)^; and another two studies^(^ 38 ^-^ 39 ^)^ evaluated the use of acupuncture for cancer pain management in adult patients with advanced cancer in palliative care.

Regarding the follow-up time, all the studies showed a short-term follow-up, with the protocols varying from a single day^(^ 36 ^,^ 40 ^)^; one week^(^ 38 ^)^; two weeks^(^ 37 ^)^, three weeks^(^ 39 ^)^, to a maximum of 4 weeks^(^ 35 ^)^.

**Figure 4:**
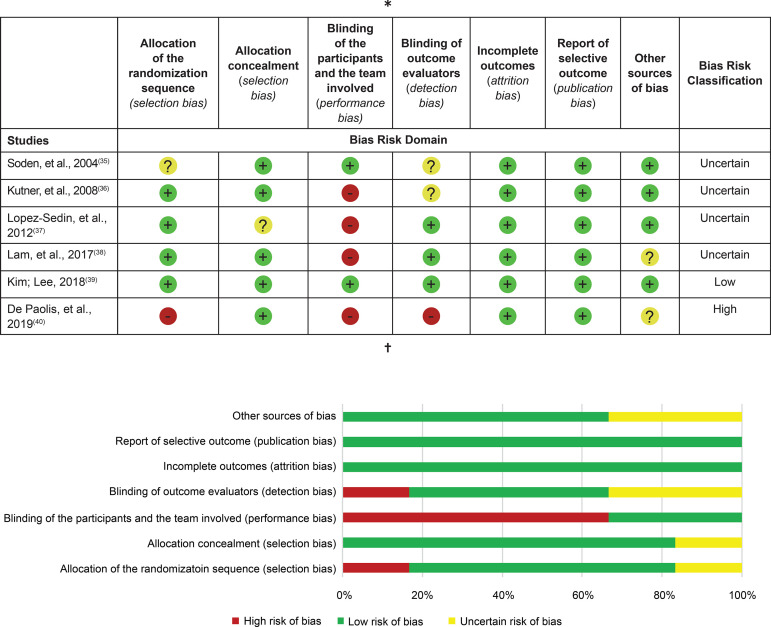
Risk for bias of the six studies included and evaluated by the Cochrane Collaboration tool^(^ 32 ^)^. Vitória, ES, Brazil, 2019 ^*^Evaluation of the internal validity and of the risk of bias of the randomized controlled trials (RCTs) included in the study according to the Cochrane Collaboration Tool to assess the risk of bias in Randomized Controlled Trials; ^†^Percentage of risk of bias among the RCTs by domains of the Cochrane Collaboration Tool to assess the risk of bias in Randomized Controlled Trials. The plus symbol (+) indicates low risk for bias; the minus symbol (-) indicates high risk for bias; the question mark (?) indicates uncertain risk for bias

With regards to the risk of bias in the studies to be selected and assessed by the Cochrane Collaboration Bias Risk tool, it was verified that, in most of them (83%), the reliability of the results can be questioned, either because they present a risk of uncertain bias (n=4; 67%)^(^ 35 ^-^ 38 ^)^ or for exhibiting a high risk of bias (n=1; 17%)^(^ 40 ^)^. Only one study was classified as being at low risk for bias, with all the domains scored in this category (Figure 4).

We observed that four of the included studies^(^ 36 ^-^ 38 ^,^ 40 ^)^, corresponding to 67% of the sample, displayed high risk for bias for the “blinding of the participants and the team involved” domain (performance bias). Two studies^(^ 35 ^-^ 36 ^)^, corresponding to 33% of the sample, displayed an uncertain risk of bias for the “blinding of outcome evaluators” domain (detection bias), and another two other^(^ 38 ^,^ 40 ^)^ also exhibited an uncertain bias risk for the “other sources of bias” domain. It should be noted that all six studies were classified as low risk for bias for the “incomplete outcomes” and “report of selective outcomes” domains, representing low attrition and publication bias, respectively.

## Discussion

The clinical use and assessment of the potential benefits of the complementary therapies in the treatment of cancer patients has recently increased in both pediatric^(^ 18 ^-^ 22 ^)^ and adult patients^(^ 23 ^-^ 26 ^)^. Among the manipulation practices based on the body, the therapeutic massage stands out as the most commonly used complementary therapy modality^(^ 41 ^-^ 42 ^)^.

In this review, half of the included studies used massage therapy as CT^(^ 35 ^-^ 37 ^)^. Another study used a combination of progressive muscle relaxation and interactive guided imaging^(^ 40 ^)^; and another two studies^(^ 38 ^-^ 39 ^)^ evaluated the use of acupuncture for the management of cancer pain in adult patients with advanced cancer in palliative care. Among the studies in this review that used massage therapy for the management of cancer pain in the study population, two demonstrated a beneficial effect^(^ 36 ^-^ 37 ^)^ and one study showed no statistically significant differences^(^ 35 ^)^.

In summary, a study^(^ 36 ^)^ suggested that the massage can be more effective than simple touch in reducing cancer pain and improving mood immediately after the treatment sessions. However, the sustained benefits of the massage in this population were less evident. Likewise, another study^(^ 37 ^)^ revealed that the combination of massage therapy and exercise showed to be effective in immediately reducing cancer pain, distress, and suffering, as well as improving mood in patients with terminal cancer.

Corroborating to the beneficial findings of the articles in the sample of our review, in other previous studies, the therapeutic massage has been shown to increase blood and lymphatic circulation, decrease inflammation and edema, relax muscles, increase dopamine and serotonin levels and also the number of lymphocytes^(^ 41 ^-^ 44 ^)^. In addition, randomized controlled trials have reported positive results from the massage therapy on the neuroendocrine and immune systems of women with early breast cancer, including reduced levels of anxiety, depression, anger, and fear, as well as increased levels of dopamine, serotonin, number of NK cells and lymphocytes. One of the mechanisms underlying for the stimulating effect on the immune system by the massage therapy probably results from the reduction of cortisol levels, which are inversely associated with the activity of NK cells, and from the increase in serotonin and dopamine levels, which lead to a reduction in cortisol release^(^ 44 ^)^.

Other research studies on massage therapy have also shown improvements in pain, nausea and other symptoms, immediately and over time^(^ 45 ^-^ 46 ^)^. The most consistent effect of massage has been to reduce the subjective degrees of anxiety, which can be more sensitive than the objective indicators for relaxation/arousal^(^ 42 ^)^. In addition, a number of qualitative studies corroborate this potential of the massage to promote relaxation and feelings of well-being^(^ 41 ^,^ 47 ^)^.

Additionally, a systematic review identified six RCTs related to the relaxing effects of aromatherapy massage. Three of these studies involved cancer patients and compared massage with and without the addition of essential oils. These studies suggest that aromatherapy massage may have a mild transient anxiolytic effect. However, there was no evidence of a sustained effect over time, and no beneficial effect on depression^(^ 48 ^)^.

Contrary to the aforementioned findings, a study in our review found that adding lavender essential oil did not appear to increase the beneficial effects of the massage^(^ 35 ^)^. In line with this finding, there is a previous study that also did not detect statistically significant changes on cancer symptoms over time^(^ 49 ^)^. A recent systematic review pointed out that, when compared to ordinary massage alone, aromatherapy massage does not provide significant effectiveness in improving anxiety among cancer patients in palliative care^(^ 50 ^)^. It should be noted that one of the main limitations in examining the effectiveness of manual massage in cancer patients is the lack of standardization of its application (technique and dosage) and the difficulty of including a control group^(^ 51 ^)^.

In our review, the results of the study that evaluated the use of MR-IGI (progressive muscle relaxation and interactive guided image) was considered as an effective adjuvant in the relief of suffering related to cancer pain in these patients^(^ 40 ^)^. In line with this result is a randomized clinical trial that evaluated the effects of muscle relaxation and guided image in 80 women with breast cancer, before and after stress periods, specifically chemotherapy, radiotherapy, and surgery. The results revealed that the use of this complementary therapy modality changed important responses of the immune system, leading to an increase in the number of activated T cells and in the NK cells’ activity^(^ 52 ^)^. A pilot RCT conducted with 40 hospitalized cancer patients who investigated the contribution of PMR + IGI to pain relief, found significant differences in pain intensity in 31% of the PMR + IGI group versus 8% in the control group^(^ 53 ^)^.

As for the studies in our review that tested the use of acupuncture^(^ 38 ^-^ 39 ^)^, they exhibited divergent results. While a study indicated that *si guanxue* acupuncture plus the commonly used acupuncture points (PC6; ST36; SP6) tends to be effective in reducing cancer pain^(^ 38 ^)^, another study pointed out that, although the treatment with IA appeared to be viable and safe for patients with advanced cancer, it did not demonstrate significant differences in the groups (experimental and control) mainly due to the control group (Sham IA) limitation^(^ 39 ^)^. A recent randomized clinical trial of parallel arms conducted with 31 cancer patients who complained of pain greater than or equal to four on the Numerical Pain Scale, and aimed to evaluate the effectiveness of auricular acupuncture on cancer pain in patients undergoing chemotherapy treatment and possible changes in the consumption of analgesics after the application of the intervention, verified that, after the eight sessions of auricular acupuncture, there was a statistically significant difference between the groups in the reduction of pain intensity (p<0.001), as well as in the consumption of medications (p<0.05). The authors concluded that auricular acupuncture was effective in reducing cancer pain in patients undergoing chemotherapy^(^ 7 ^)^.

Moreover, a review of the literature reported diverse evidence that acupuncture improves the immune function through the modulation of the NK cells’ activity. A hypothetical model has been proposed to explain how acupuncture stimulates the immune system by stimulating the ST36 acupoint. This point is known as the “immune boosting point”, as it is able to improve the functioning of the immune system. The stimulation of this acupoint induces the release of nitric oxide, a neurotransmitter that stimulates, through sensory nerves, the lateral area of the hypothalamus, promoting the secretion of opioid peptides, such as β-endorphin. Through the bloodstream, this peptide reaches the spleen and other parts of the body, binding to the opioid receptors expressed on the surface of NK cells. When binding to the receptors, β-endorphin stimulates the NK cells to amplify the expression of cytotoxic molecules, tumoricidal activity and, consequently, the production of IFN-γ. This cytokine induces the expression of NK cell receptors and possibly the secretion of cytokines by other cells of the immune system, orchestrating and amplifying anti-cancer immune responses^(^ 54 ^)^.

Acupuncture is one of the most popular forms of complementary medicine^(^ 29 ^,^ 55 ^)^ and its use is mainly linked to improving the psychological symptoms through sympathomimetic pathways^(^ 56 ^)^. Traditional Chinese Acupuncture (TCA) is used as a complement to the conventional treatment for several pathological conditions and its focus is to relieve symptoms by reorganizing the body’s energy, aiming at leading to self-healing^(^ 55 ^)^. Sham Acupuncture (SA), also called placebo, can be understood as an intervention performed in a false way, as it is performed outside the points established by the TCA^(^ 57 ^)^. The scarcity of research studies with acceptable controls that actually mimic all aspects of the tested intervention has been the main methodological problem presented by the studies that use acupuncture as a therapy^(^ 29 ^,^ 57 ^)^.

This systematic review has some limitations. When evaluated methodologically by the Cochrane Collaboration tool, most of the included studies displayed a risk for uncertain bias (n=4; 67%), leading to questions about the reliability of the results, thus compromising the external validity of these studies. Another important limitation concerns the fact that different interventions are being evaluated in different types of cancer, making the studies heterogeneous and, for this reason, quantitative assessments were not feasible. In addition, the short follow-up time (follow-up in a single day and up to a maximum of four weeks) may have impaired the measurement of some outcomes. To this end, it is suggested that new RCTs be conducted with a longer follow-up, to detect whether the effects of using complementary therapies for cancer pain management in these patients are sustained in the medium and long term. Thus, there is a need for further RCTs with representative samples of the population and with low risk for bias.

## Conclusion

The evidence from these six RCTs, mainly in three studies that evaluated the use of the massage therapy for cancer pain management in adults with cancer in palliative care, showed to be effective and promising for pain reduction. However, although the three studies that addressed massage therapies have positive results and the qualitative analysis of the review suggests the benefit of this practice in reducing cancer pain, the need for further studies with representative samples and rigorous methodological designs is highlighted in order to confirm such findings, since the three studies were evaluated with uncertain bias risk. Due to the fact that they exhibit opposite results, the two studies that evaluated the use of acupuncture as a complementary therapy were insufficient to accurately assert the efficacy of such therapy on the reduction of cancer pain, mainly because they differ on the methodological aspects (type of acupuncture, application techniques, and evaluated acupuncture points), therefore needing to get more evidence to elucidate such findings.
